# Reduced sleep efficiency, measured using an objective device, was related to an increased prevalence of home hypertension in Japanese adults

**DOI:** 10.1038/s41440-019-0329-0

**Published:** 2019-09-18

**Authors:** Takumi Hirata, Tomohiro Nakamura, Mana Kogure, Naho Tsuchiya, Akira Narita, Ken Miyagawa, Kotaro Nochioka, Akira Uruno, Taku Obara, Naoki Nakaya, Hirohito Metoki, Masahiro Kikuya, Junichi Sugawara, Shinichi Kuriyama, Ichiro Tsuji, Shigeo Kure, Atsushi Hozawa

**Affiliations:** 1 Tohoku Medical Megabank Organization, Tohoku University, Sendai, Japan; 2 Graduate School of Medicine, Tohoku University, Sendai, Japan; 3 OMRON Healthcare Co., Ltd., Muko, Japan; 4 Tohoku University Hospital, Tohoku University, Sendai, Japan; 5 School of Medicine, Tohoku Medical and Pharmaceutical University, Sendai, Japan; 6 Teikyo University School of Medicine, Tokyo, Japan; 7 International Research Institute of Disaster Science, Tohoku University, Sendai, Japan

**Keywords:** Sleep efficiency, Contactless biomotion sensor, Home blood pressure, Epidemiology

## Abstract

Few studies have reported the relationship between reduced sleep efficiency and the prevalence of hypertension independent of sleep duration in Japan. This study aimed to evaluate whether reduced sleep efficiency, measured using an objective device for >1 week, was related to an increased prevalence of hypertension independent of sleep duration in the general Japanese population. We conducted a cross-sectional study of 904 participants aged ≥20 years who lived in Miyagi Prefecture, Japan. Sleep efficiency was measured using a contactless biomotion sleep sensor for 10 continuous days. The participants were classified into two groups according to their sleep efficiency: reduced (<90%) or not reduced (≥90%). Hypertension was defined as morning home blood pressure ≥135/85 mmHg or self-reported treatment for hypertension. Multivariable logistic regression models were used to obtain odds ratios (ORs) and 95% confidence intervals (CIs) to assess the relationship between sleep efficiency and hypertension adjusted for potential confounders. The results showed that two hundred and ninety-four individuals (32.5%) had reduced sleep efficiency, and 331 (36.6%) had hypertension. Individuals with reduced sleep efficiency had a higher body mass index and shorter sleep duration. In the multivariable analysis, reduced sleep efficiency was significantly related to an increased prevalence of hypertension (OR, 1.62; 95% CI, 1.15–2.28). In conclusion, reduced sleep efficiency was significantly related to an increased prevalence of hypertension in Japanese adults. Improvements in sleep efficiency may be important to reduce blood pressure in Japanese adults.

## Introduction

Short sleep duration is related to an increased prevalence or incidence of hypertension [1–3]. In most of these studies, sleep duration was measured using self-reported questionnaires. However, self-reported sleep duration does not accurately reflect actual sleep duration because studies have shown inadequate correlations between self-reported sleep duration and actigraphy-measured sleep duration [4, 5]. A few recent epidemiological studies in a Japanese population have reported the relationships among actigraphy-measured sleep duration, an objective indicator, and health outcomes [6, 7]. In these studies, however, the relationship between objectively measured sleep duration and hypertension was insufficiently examined.

Recently, some studies in the United States have reported that reduced sleep efficiency and short sleep duration were related to an increased prevalence of hypertension [8, 9]. Reduced sleep efficiency, but not sleep duration, was particularly related to an increased prevalence of hypertension independent of sleep-disordered breathing in a large sample of Hispanic/Latino adults [8]. The findings suggest that sleep efficiency, but not sleep duration, is an important target to maintain good cardiovascular health. In Japan, one study revealed that reduced sleep efficiency measured using actigraphy had higher nighttime blood pressure in elderly participants [10]; however, these researchers did not evaluate whether the relationship between reduced sleep efficiency and prevalent hypertension was independent of sleep duration. In addition, no previous reports have revealed the relationship between hypertension and sleep efficiency measured for >1 week.

Therefore, this study aimed to evaluate whether reduced sleep efficiency measured using an objective sleep monitoring device for >1 week was related to an increased prevalence of hypertension independent of sleep duration in the general Japanese population.

## Methods

### Participants

This cross-sectional study was conducted to clarify the relationship between sleep efficiency and hypertension. Participants fulfilling the following criteria were included in this study: (1) participants receiving the follow-up survey from the Tohoku Medical Megabank Project Cohort Study (TMM Cohort Study), (2) participants aged ≥20 years living in Miyagi Prefecture during the baseline survey from May 2013 to March 2016, and (3) participants who visited one community support center (located in Tagajo City in Miyagi Prefecture). The TMM Cohort Study was a population-based prospective cohort study that has been ongoing since 2013. All participants were recruited from June 2017 to March 2018. Written informed consent was obtained from each participant. This study was approved by the Institutional Review Board of the Tohoku Medical Megabank Organization (approval number: 2017-4-007).

In total, 1500 participants were initially included. However, 235 participants were excluded for the following reasons: (1) home blood pressure was measured for <3 days (*n* = 75); (2) sleep efficiency or sleep duration was measured for <7 days (*n* = 375); (3) urinary sodium/potassium ratio was measured for <10 days (*n* = 130); (4) daily steps were measured for <3 days (*n* = 12); and (5) data on sex, age, body weight, height, alcohol drinking status, and smoking status were missing (*n* = 4). Finally, we analyzed the data for 904 participants.

### Measurements

All participants completed a self-reported questionnaire to assess demographic characteristics, smoking status, alcohol drinking status, information on treatment for hypertension and diabetes, and history of cardiovascular diseases. Height and weight were measured with all participants wearing light clothing. The body mass index (BMI) was calculated as the weight (kg) divided by the square of the height (m). Obesity was defined as BMI ≥ 25 kg/m^2^ based on the Western Pacific Region of WHO criteria for Japanese individuals [11]. Blood samples were obtained from nonfasting participants. Plasma glucose concentrations and HbA1c levels were measured using an enzymatic method. The presence of diabetes was defined as plasma glucose ≥200 mg/dL and/or HbA1c ≥6.5% and/or receiving treatment for diabetes.

Home blood pressure was assessed using a cuff-oscillometric device (HEM-7080IC; Omron Healthcare Co., Ltd, Kyoto, Japan). Home blood pressure was recorded for 10 days in a sitting position after at least 5 min of rest in the morning within 1 h after awakening, maintaining the arm-cuff position at heart level during resting, and if applicable, before taking medications for hypertension, eating breakfast, and after urination. We defined morning home blood pressure as the mean of the first measurement performed every morning. Hypertension, the primary outcome of the study, was defined as morning home blood pressure ≥135/85 mmHg or receiving treatment for hypertension [12].

Sleep efficiency (main exposure) and sleep duration were measured using a contactless biomotion sleep sensor (HSL-102 M; Omron Healthcare Co., Ltd) for 10 days. The details of measurement using a sleep sensor have been described elsewhere [13–15]. The definition of sleep efficiency was the ratio of the sleep duration (min) to the time spent in bed (min) multiplied by 100 [16]. We used the average of the data for all measurements in the analysis. All participants were classified into two groups according to their sleep efficiency (reduced; <90%/not reduced; ≥90%).

The urinary sodium to potassium ratio was measured using a hand-sized urinary Na/K ratio monitor (HEU-001F; Omron Healthcare Co., Ltd.) for 10 continuous days. The urinary sodium to potassium ratio was measured twice per day (in the morning after awakening and at night before going to bed). We used the average of the data for all measurements in the analysis.

The daily steps were counted using an activity monitor (HJA-750C; Omron Healthcare Co., Ltd). The activity monitor was positioned at the waist of each participant and was attached throughout the day, except while bathing and sleeping for 11 continuous days. Of the 11 days, the data from the 1st day were excluded. In addition, we excluded the data when the daily total measurement duration was <8 h. We used the average of the data for all measurements for this analysis.

### Statistical analysis

The data were presented as the means (standard deviations) or medians (interquartile ranges) for continuous variables or numbers (percentages) for categorical variables. We used an unpaired *t*-test or the Wilcoxon rank-sum test for continuous variables and the chi-square test or Fisher’s exact test for categorical variables to compare the characteristics between the two groups. Multivariable logistic regression models were used to obtain odds ratios (ORs) and 95% confidence intervals (CIs) to assess the relationship between sleep efficiency and hypertension. The models were adjusted for sex, age, BMI, alcohol drinking status, smoking status, average daily steps, urinary sodium/potassium ratio, presence of cardiovascular diseases, presence of diabetes, and sleep duration. Similarly, we analyzed the relationship between sleep efficiency and hypertension if the definition of reduced sleep efficiency was <85%. We also used multivariable regression analysis to assess the relationship between sleep efficiency and home blood pressure. In addition, we analyzed the impact of sleep duration and obesity on the relationship between sleep efficiency and the prevalence of hypertension. We divided all participants into four groups according to the presence of reduced sleep efficiency and shorter sleep duration or obesity. Thereafter, we used a multivariable logistic regression model to assess the relationship between each combination and the prevalence of hypertension. Two-tailed *P* values of <0.05 were considered statistically significant. All analyses were performed using STATA SE 13 data analysis and statistical software (Stata Corp LP, College Station, TX, USA).

## Results

**Fig. 1:**
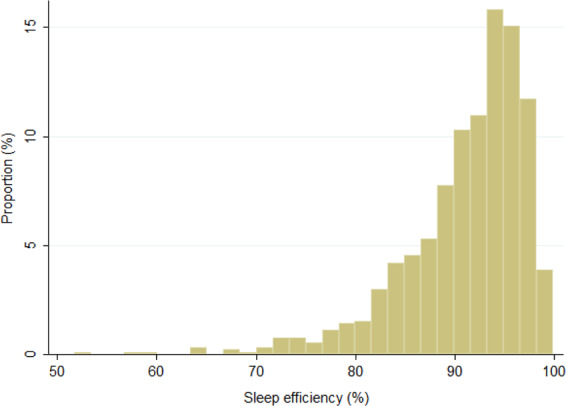
Distribution of sleep efficiency among all participants

The clinical characteristics of all participants according to sleep efficiency are presented in Table 1. Two hundred ninety-four participants (32.5% of all participants) had reduced sleep efficiency, and 331 participants (36.6%) had hypertension. The distribution of sleep efficiency among all participants is depicted in Fig. 1. The prevalence of reduced sleep efficiency was significantly higher in men (40.9% in men vs. 28.8% in women), and participants with reduced sleep efficiency had significantly higher BMI (23.4 vs. 22.3 kg/m^2^), shorter sleep duration (5.9 vs. 6.4 h/day), a higher urinary sodium/potassium ratio (4.4 vs. 4.2), and higher prevalence of hypertension (44.9% vs. 32.6%) compared with those without reduced sleep efficiency.

**Table 1:** Clinical characteristics of all participants according to sleep efficiency

	Sleep efficiency		*P*-value
	Reduced (<90%)	Not reduced (≥90%)	
Participants, *n*	294	610	
Sleep efficiency (%)	83.9 (5.9)	94.6 (2.4)	<0.001
Sex, male	114 (38.8%)	165 (27.1%)	<0.001
Age (years)	58.1 (14.7)	60.4 (12.3)	0.173
Body mass index (kg/m^2^)	23.4 (3.5)	22.3 (3.0)	<0.001
Current drinker, yes	157 (53.4%)	307 (50.3%)	0.386
Current smoker, yes	20 (6.8%)	39 (6.4%)	0.815
Urinary Na/K ratio	4.4 (1.5)	4.2 (1.6)	0.045
Sleep duration (h/day)	5.9 (1.1)	6.4 (1.1)	<0.001
Steps (10^3^ steps/day)	6.0 (4.5–7.8)	6.1 (4.4–7.8)	0.904
Cardiovascular disease, yes	19 (6.5%)	31 (5.1%)	0.395
Diabetes, yes	25 (8.5%)	38 (6.2%)	0.208
Hypertension, yes	132 (44.9%)	199 (32.6%)	<0.001
Home systolic BP (mmHg)	127.7 (17.2)	123.0 (15.3)	<0.001
Home diastolic BP (mmHg)	76.1 (10.2)	73.6 (9.4)	<0.001
Treatment for hypertension, yes	66 (22.5%)	97 (15.9%)	0.016

*Na*/*K* sodium/potassium, *BP* blood pressure

The results of the relationship between sleep efficiency and the prevalence of hypertension are presented in Table 2. In the univariate analysis, reduced sleep efficiency was significantly related to an increased prevalence of hypertension (OR, 1.68; 95% CI, 1.27–2.24). In addition, male sex, older age, higher BMI, current alcohol drinking, lower average daily steps, presence of cardiovascular disease, and presence of diabetes were significantly related to an increased prevalence of hypertension. In multivariable analysis, reduced sleep efficiency was still significantly related to an increased prevalence of hypertension (OR, 1.62; 95% CI, 1.15–2.28) adjusted for all potential confounders, including sleep duration. When we excluded sleep duration from the multivariable model, the relationship between reduced sleep efficiency and the prevalence of hypertension remained unchanged (OR, 1.65; 95% CI, 1.18–2.31). When we defined reduced sleep efficiency <85%, the OR (95% CI) of participants with reduced sleep efficiency was 1.92 (1.21–3.04) in multivariable analysis. In addition, log-transformed sleep efficiency was significantly inversely related to home systolic blood pressure (*β* = −27.39, *p* < 0.001) or home diastolic blood pressure (*β* = −12.91, *p* = 0.007) adjusted for potential confounders in participants who did not receive treatment for hypertension.

**Table 2:** Association between sleep efficiency and hypertension

	Univariable model		Multivariable model	
	OR	95% CI	OR	95% CI
Reduced sleep efficiency (<90%)	1.68	(1.27, 2.24)	1.62	(1.15, 2.28)
Sex, male	3.19	(2.38, 4.28)	1.33	(0.92, 1.94)
Age (per year)	1.07	(1.06, 1.09)	1.07	(1.05, 1.09)
BMI (per kg/m^2^)	1.20	(1.15, 1.26)	1.16	(1.10, 1.22)
Current drinker, yes	1.59	(1.21, 2.09)	1.50	(1.07, 2.09)
Current smoker, yes	1.20	(0.70, 2.06)	1.62	(0.86, 3.05)
Urinary Na/K ratio	1.08	(0.99, 1.17)	1.13	(1.02, 1.24)
Steps per day^a^	0.68	(0.50, 0.91)	0.79	(0.56, 1.11)
Cardiovascular disease, yes	2.52	(1.41, 4.50)	1.23	(0.64, 2.33)
Diabetes, yes	2.84	(1.68, 4.80)	1.41	(0.80, 2.51)
Sleep duration (per hour)	1.10	(0.98, 1.25)	0.95	(0.82, 1.11)

Multivariable model was adjusted for sex, age, body mass index, alcohol drinking status, smoking status, average daily steps, urinary sodium/potassium ratio, cardiovascular disease, diabetes, and sleep duration

*OR* odds ratio, *CI* confidence interval, *PAF* population attributable fraction, *BMI* body mass index, *Na*/*K* sodium/potassium

^a^log-transformed

Finally, we present the impact of sleep duration and obesity on the relationship between sleep efficiency and the prevalence of hypertension in Table 3. Short sleepers with reduced sleep efficiency were significantly related to an increased prevalence of hypertension compared with nonshort sleepers without reduced sleep efficiency adjusted for potential confounders (OR, 1.96; 95% CI, 1.23–3.13; Table 3a). In addition, obesity and a reduction in sleep efficiency were additively related to an increased prevalence of hypertension. The ORs (95% CIs) of obese participants without reduced sleep efficiency, nonobese participants with reduced sleep efficiency, and obese participants with reduced sleep efficiency were 2.67 (1.65–4.31), 1.64 (1.11–2.42), and 5.28 (2.95–9.45), respectively (Table 3b).

**Table 3:** The impact of short sleeping duration and overweight on the association between sleep efficiency and hypertension

	OR	95% CI
A. Presence of short sleep duration (<6 h)		
Not reduced sleep efficiency and nonshort sleeper (*n* = 410)	Ref.	
Not reduced sleep efficiency and short sleeper (*n* = 200)	1.02	(0.67, 1.56)
Reduced sleep efficiency and nonshort sleeper (*n* = 141)	1.43	(0.92, 2.23)
Reduced sleep efficiency and short sleeper (*n* = 153)	1.96	(1.23, 3.13)
B. Presence of obesity (BMI ≥25 kg/m^2^)		
Not reduced sleep efficiency and nonobese (*n* = 505)	Ref.	
Not reduced sleep efficiency and obese (*n* = 105)	2.67	(1.65, 4.31)
Reduced sleep efficiency and nonobese (*n* = 212)	1.64	(1.11, 2.42)
Reduced sleep efficiency and obese (*n* = 82)	5.28	(2.95, 9.45)

Reduced sleep efficiency defined as sleep efficiency  90%

Model A was adjusted for sex, age, BMI, alcohol drinking status, smoking status, average daily steps, urinary sodium/potassium ratio, cardiovascular disease, and diabetes

Model B was adjusted for sex, age, alcohol drinking status, smoking status, average daily steps, urinary sodium/potassium ratio, cardiovascular disease, diabetes, and sleep duration

*OR* odds ratio, *CI* confidence interval, *BMI* body mass index

## Discussion

We showed that reduced sleep efficiency (<90%) measured using a contactless biomotion sleep sensor was significantly related to an increased prevalence of hypertension independent of sleep duration in Japanese adults in the present study. In particular, short sleep duration with poor sleep efficiency was significantly related to an increased prevalence of hypertension. In addition, reduced sleep efficiency was significantly related to hypertension in both obese and nonobese participants.

Reduced sleep efficiency or reduced sleep continuity, but not sleep duration, was related to an increased prevalence of hypertension in several previous studies in the United States of America [8, 17], and our findings were consistent with these studies. The mechanism of the relationship between sleep efficiency and hypertension may be explained by an increased sympathetic tone. Sleep deprivation increases sympathetic tone [18–20]. Similarly, poor sleep quality is related to the lack of nocturnal dip in blood pressure due to increasing nocturnal sympathetic activity [21, 22]. Thus, our findings suggest that sleep efficiency is an important target to maintain good control of blood pressure.

In the additional analyses of the impact of obesity on the relationship between sleep efficiency and the prevalence of hypertension, we showed that obesity and reduced sleep efficiency were additively related to an increased prevalence of hypertension. Reduced sleep efficiency was significantly related to cardiovascular-related diseases, including hypertension among obese Asian patients with obstructive sleep apnea [23]. However, in the present study, reduced sleep efficiency was significantly related to an increased prevalence of hypertension, even in participants who were not obese. Our findings indicate that improved sleep efficiency might be important to achieve good blood pressure control, regardless of obesity.

This study had several strengths. First, our investigation of the relationship between objective sleep efficiency and the prevalence of hypertension persisted for >1 week. Several epidemiological studies have shown a relationship between objective sleep efficiency and hypertension using actigraphy or polysomnography; however, the measurement period was <1 week in most of the studies. Second, we used a blood pressure of ≥135/85 mmHg measured at home as the definition of hypertension. Participants with masked hypertension, which is an important risk factor for cardiovascular disease, could be identified by home blood pressure monitoring. We showed for the first time that sleep efficiency made a substantial contribution to prevalent hypertension.

In contrast, this study had several limitations. First, the study was cross-sectional, and thus inferences regarding the direction of relationships and/or causality were not possible. Therefore, prospective cohort studies are required to clarify the causal relationship. Second, the impact of sleep-disordered breathing, including insomnia and sleep apnea, could not be evaluated because we could not monitor sleep-disordered breathing. Sleep-disorder breathing has been shown to be related to hypertension [21, 24] or blood pressure variability [25], and a nondipping pattern in normotensive patients with obstructive sleep apnea may progress to carotid atherosclerosis [26]. Our findings may partially represent the relationship between sleep-disordered breathing and hypertension. Third, we could not measure several comorbidities, including restless leg syndrome, and thus, the impact of these comorbidities on the relationship could not be evaluated. Finally, we defined hypertension based on home BP, not office BP, in the present study because home BP is superior to office measurement as a predictor of cardiovascular risk in previous studies [27, 28]. However, the impact on office BP, including masked hypertension, should be shown, and thus, we will examine the impact on office BP in the future.

In conclusion, reduced sleep efficiency was significantly related to an increased prevalence of hypertension independent of sleep duration and BMI in Japanese adults in the present study. Sleep efficiency could be improved by the maintenance of the living environment related to sleep and improvements in lifestyle, including exercise habits. We suggest that sleep efficiency may be an important target to prevent hypertension in the Japanese population, and we anticipate the development of an effective method for improving sleep efficiency in the future.
